# High Nationwide Incidence of Multiple Sclerosis in Sweden

**DOI:** 10.1371/journal.pone.0108599

**Published:** 2014-09-29

**Authors:** Cecilia Ahlgren, Anders Odén, Jan Lycke

**Affiliations:** 1 Department of Clinical Neuroscience and Rehabilitation, Institute of Neuroscience and Physiology, The Sahlgrenska Academy, University of Gothenburg, Gothenburg, Sweden; 2 Institute of Mathematical Statistics, Department of Mathematical Sciences, Chalmers University of Technology, Gothenburg, Sweden; Federal University of Rio de Janeiro, Brazil

## Abstract

Over recent years increased MS incidence, primarily in women, has been reported. We recently reported an unexpectedly high MS prevalence of 189/100,000 in Sweden. In the present study we estimated the nationwide age- and gender-specific MS incidence and the sex ratio in Sweden between 2001 and 2008. MS patients were identified by linking two nationwide health data registers, and the Swedish population register. The earliest registered date of MS diagnosis was determined. By logistic regression, the probability of the date of MS diagnosis being within the incidence period, depending on age and time was estimated for a subset of patients and applied to other patients. By Poisson regression, the hazard functions for the incidence of MS diagnosis were estimated. The expected number of MS patients was 7,361.4. The incidence in the average population of 9,054,658 was 10.2 per 100,000 person-years, and 6.2 and 14.0 per 100,000 person-years for men and women, respectively. The crude female to male ratio was 2.26. No increase of incidence or change of sex ratio was observed from 2001 to 2008. In conclusion, the average MS incidence in Sweden from 2001 to 2008 was 10.2 per 100.000, which was considerably higher than previous regional Swedish estimates of 4.3–6.4. No increase of female to male ratio of MS during the study period was observed. We provide supplementary data that can be used as tools for examining excess MS risk in different study materials.

## Introduction

Several studies [1]–[5] and systematic reviews [6], [7] report increased incidence of MS, primarily in women. We recently reported the 2008 nationwide gender- and age-specific prevalence of MS in Sweden. It was unexpectedly high (188.9 per 100,000, 113.4 for men and 263.6 for women) [8], and considerably higher than previous regional estimates [9]–[11]. However, besides the true change of MS frequency, higher MS prevalence in current compared with previous investigations may be explained by several other factors such as: improved case ascertainment, changed diagnostic criteria, better awareness of MS, improved healthcare services and socioeconomic conditions and extended survival time. Although MS incidence is not affected by the survival time and better reflect MS risk, the number of cases and the observational time are often limited, making incidence estimates statistically uncertain. Over recent years, age- and gender-specific MS incidence in populations larger than one million have been reported from Uusimaa, Finland [12], Olmsted County in the US [13], the UK [14], Taiwan [15], and France [16]. However, investigating MS incidence on a large scale, involves other challenging problems which are related to the difficulties in achieving complete ascertainment of patients, obtaining information about age and residency at the time of disease onset or diagnosis, and obtaining age- and gender-specific population data for each calendar year.

Sweden, with a population of approximately 9 million, has several important preconditions for nationwide investigations of disease; individual demographical information about all Swedish residents, mandatory registration of diagnoses in a nationwide health data register as well as nationwide disease-specific registers, a 10-digit personal identity number which allows linkage between different Swedish registers, and yearly age- and gender-specific population data. In previous regional Swedish estimates the MS incidence was 4.3 in Gothenburg city (1984–1988) [11], 5.2 in Västerbotten county (1988–1997) [10], and 6.4 in Värmland county (1991–2000) [9]. In the present study, we estimated the nationwide age-and gender-specific incidence and sex ratio of MS in Sweden from 2001 to 2008.

## Materials and Methods

### Area and population

Sweden lies between latitudes 55° and 69° north in Northern Europe. Despite its northern latitude, Sweden has a temperate climate. The mean temperature is between −16°C and ±0°C in January and +8°C and +16°C in July (http://www.smhi.se). The population of Sweden increased from 8,909,128 in 2001 to 9,256,347 in 2008 (average 9,054,657.6). The population density is low at 23 inhabitants per square kilometre, and 85% of the population lives in urban areas (http://www.scb.se). In 2008, the mean age of the Swedish population was 41 years, the birth rate was 12/1000, the mortality rate was 10/1000, the immigration rate was 11/1000, and 5/1000 persons emigrated. At the end of 2008, approximately 14% of the population of Sweden were born abroad. Twenty-one percent of the immigrant population was born in other Nordic countries, 36% in non-Nordic European countries, and the remaining 43% in non-European countries (http://www.scb.se).

### Ethics approval

All individual data from the different sources were made anonymous to the authors by the replacement of the personal identity numbers by unique number codes for use in the present study. Thus, informed consent was not obtained. The study was approved by the regional ethical review board in Gothenburg.

### Swedish national health and population registers

The collection of information on patients at public hospitals in the Swedish National Patient Register (NPR) started in the 1960s. Data entered into the NPR is mandatory and prospective (http://www.socialstyrelsen.se). The register consists of two arms: the inpatient care register (Inpatient NPR), complete since 1987; and the outpatient care register (Outpatient NPR), covering clinical visits since 2001. Chief information includes the personal identity number, gender, date of birth, and every clinical visit, hospital admission, or discharge correlated with principal and secondary diagnoses. The NPR was searched for the dates of all clinical visits or discharges registered with a principal or secondary diagnosis of MS, which was coded as G359, 340, or 340.99 according to the International Classification of Diseases (ICD) 10, 9, or 8, respectively.

The nationwide Swedish Multiple Sclerosis Registry (SMSreg) (http://www.msreg.net) began in 1996. It is now maintained by the Swedish Association of Local Authorities and Regions (http://www.skl.se) and the National Board of Health and Welfare (http://www.socialstyrelsen.se). The SMSreg is web-based, allowing updates of individual data during clinical visits. Data are mainly prospectively, but also retrospectively entered. The core data set includes the personal identity number, date of birth, gender, age, date of onset, date of diagnosis, disease course, and diagnostic investigations such as MRI and CSF examinations. At the end of 2008, approximately 11,000 MS patients were registered in the SMSreg and the coverage was 60% [8]. The SMSreg was searched for the date of diagnosis of MS or possible MS [17]–[19].

Since 1947, every individual who has resided in Sweden on a permanent basis has been mandatorily assigned a 10-digit unique personal identity number *in the Swedish Total Population Register* (TPR). For these individuals, the property unit in the parish is registered each year and at the time of moving or migration. Only a small number of individuals living in Sweden do not have a Swedish personal identity number (mainly immigrants waiting for a residence permit). These persons were not included in the patient or general population figures in the present study. Besides data on residence, crucial information includes date of birth and death, and country of birth. The TPR was established by Statistics Sweden (http://www.scb.se). This register was searched for individual information about residence at different time points. Statistics Sweden provided gender-specific population data from the TPR in one-year age groups for each calendar year from 1968 through 2008 (http://www.scb.se).

### Statistical analyses

#### The date of diagnosis

The MS incidence period was 1 January 2001 through 31 December 2008. This starting point was chosen because the Outpatient NPR started 2001, which resulted in an almost complete registration of both inpatient and outpatient MS care from this year onward. Data retrieved from the Inpatient and Outpatient NPR included unique patients diagnosed with MS at the latest on 31 December 2008. Data retrieved from the SMSreg on the 24 March 2010 encompassed MS diagnoses registered at the latest in 2009 or 2010. In total, 26,738 unique MS patients were affiliated with the NPR and/or the SMSreg. Of these, 15,265 patients were affiliated with the NPR only. The 11,473 MS patients in the SMSreg were divided into 7 disjoint subsets with respect to the registered date of MS diagnosis (A, B, C, D, E, F, and G). Subset G was an exception because it comprised patients who were included in the SMSreg only, and had no registered date of diagnosis. The expected number of patients in subset G was estimated from the proportions of male and female patients with the earliest date of diagnosis before, during, and after the incidence periods in E and F. The remaining patients, those affiliated with the NPR only, were divided into 2 disjoint subsets with respect to the earliest registered date of MS diagnosis (H and I, Table 1). Thus, by these procedures we identified all Swedish patients with a diagnosis of MS with the earliest registered date of MS diagnosis within the incidence period between 2001 and 2008.

**Table 1:** Disjoint subsets of MS patients with respect to the earliest date of MS diagnosis registered in the Swedish National Patient Register (NPR) and/or the Swedish Multiple Sclerosis Registry (SMSreg).

Subset^a^	Number of patients	NPR The earliest date of diagnosis 2001 to 2008	SMSreg The date of diagnosis 2001 to 2008	Probability of the earliest date of diagnosis 2001 to 2008	Expected number of patients contributing to the incidence 2001 to 2008
**A**	5,241	Yes	Yes, or earlier	0 or 1^b^	3446
**B**	1,924	No, earlier	Yes, or earlier	0	0^c^
**C**	1,983	Yes	*Missing*	0–1	1179.3
**D**	1,485	No, earlier	*Missing*	0	0^c^
**E**	369	*Missing*	Yes or later^d^	0 or 1^b^	71
**F**	75	*Missing*	No, earlier	0	—
**G**	396	*Missing*	*Missing*	NA	68.8^e^
**H**	4,044	Yes	NA	0–1	2596.3
**I**	11,221	No, earlier	NA	0	0^c^

a A–G were affiliated with the SMSreg only or both registers, and H and I were affiliated with the NPR only.

b The probability was 0 or 1 depending on whether the date of diagnosis in the SMSreg was from 2001 to 2008.

c The earliest date of diagnosis could not have occurred between 2001 and 2008 due to an earlier date in the NPR.

d The date of diagnosis in the SMSreg could be 2009 or 2010.

e Estimated from E and F.

NPR, Swedish National Patient Register; SMSreg, Swedish Multiple Sclerosis Registry; NA, not applicable.

#### Logistic regression analysis

During the incidence period from 2001 to 2008, virtually every in- and outpatient care visit was registered in the NPR with a diagnosis and date. Because the outpatient NPR began 2001, an MS diagnosis could have occurred earlier but was registered for the first occasion during the incidence period. To overcome this problem, we used the subset A of patients for whom we had information from both the NPR and the SMSreg. It consisted of patients with a date of diagnosis within the incidence period in the NPR and, in addition, a date of diagnosis in the SMSreg. This subset was analysed by logistic regression (Supplement S1). The probability that the first registered date of diagnosis within the incidence period reflected the real date of diagnosis was calculated as a function of age at the earliest date of diagnosis and time elapsed since 1 January 2001. Whether or not the date of diagnosis in the SMSreg was earlier than 1 January 2001 was a dependent, 0/1 variable in the analysis. Three functions of age and 4 functions of time since the beginning of 2001 were independent variables. The probability estimates from A were then applied to patients of the other subsets (Table 1). Thus, instead of following a simple rule for the date of diagnosis, we applied the individually calculated probabilities for the next step, the Poisson regression analysis. The expected numbers given by 1 decimal of the right column of table 1 were determined by use of the logistic regression model. When there was no decimal the numbers were exactly determined from the material.

#### Poisson regression analysis

A hazard function for the incidence of MS diagnosis depending on age was estimated for each gender by Poisson regression analysis. These hazard functions were determined for the population of Sweden from 2001 to 2008. The age-specific hazard function was equal to age-specific incidence. Notably, patients with probability 1 were included along with patients with lower probability in the Poisson regression analysis (Table 1). We included the general population of Sweden delineated by gender and age in one-year age groups as well as MS patients in the Poisson regression. Spline functions were used to achieve continuous and smooth curves. From β coefficients (Supplement S1), yielded by Poisson regression, the expected numbers of patients with diagnosis of MS during different years were calculated.

## Results

### Nationwide MS incidence 2001–2008 in Sweden

The overall annual incidence of MS diagnosis in Sweden during the period from 1 January 2001 through 31 December 2008 was 10.2 per 100,000 person-years for both genders combined, based on the expected number of 7,361.4 patients in the average population of 9,054,658 persons in Sweden. For men the incidence was 6.2 per 100,000 based on the expected number of 2,241.6 patients in the average population of 4,490,778 persons, and for women it was 14.0 per 100,000 based on the expected number of 5,119.8 patients in the average population of 4,563,880 persons. Except for 2001 the incidence of MS seemed essentially stable during the incidence period (Table 2).

**Table 2:** Incidence of MS diagnosis by calendar year.

	Males			Females			Both genders			
Year	MS	Population N	Incidence	MS	Population N	Incidence	MS	Population N	Incidence	Sex ratio
**2001**	**198,6**	4408445	**4,5**	**477,3**	4500683	**10,6**	**675,9**	8909128	**7,6**	**2,36**
**2002**	**247,2**	4427107	**5,6**	**646,5**	4513681	**14,3**	**893,7**	8940788	**10,0**	**2,70**
**2003**	**271,6**	4446656	**6,1**	**660,3**	4529014	**14,6**	**931,9**	8975670	**10,4**	**2,39**
**2004**	**304,0**	4466311	**6,8**	**664,6**	4545081	**14,6**	**968,6**	9011392	**10,7**	**2,15**
**2005**	**327,1**	4486550	**7,3**	**660,1**	4561202	**14,5**	**987,2**	9047752	**10,9**	**1,99**
**2006**	**273,4**	4523523	**6,0**	**638,8**	4589734	**13,9**	**912,2**	9113257	**10,0**	**2,32**
**2007**	**310,5**	4563921	**6,8**	**683,8**	4619006	**14,8**	**994,3**	9182927	**10,8**	**2,18**
**2008**	**316,5**	4603710	**6,9**	**707,6**	4652637	**15,2**	**1024,1**	9256347	**11,1**	**2,20**

### Sex ratio

The overall female to male ratio was 2.26, range 1.99–2.70 (Table 2). There was no increase or trend in sex ratio during the incidence period.

### Age distribution

**Figure 1:**
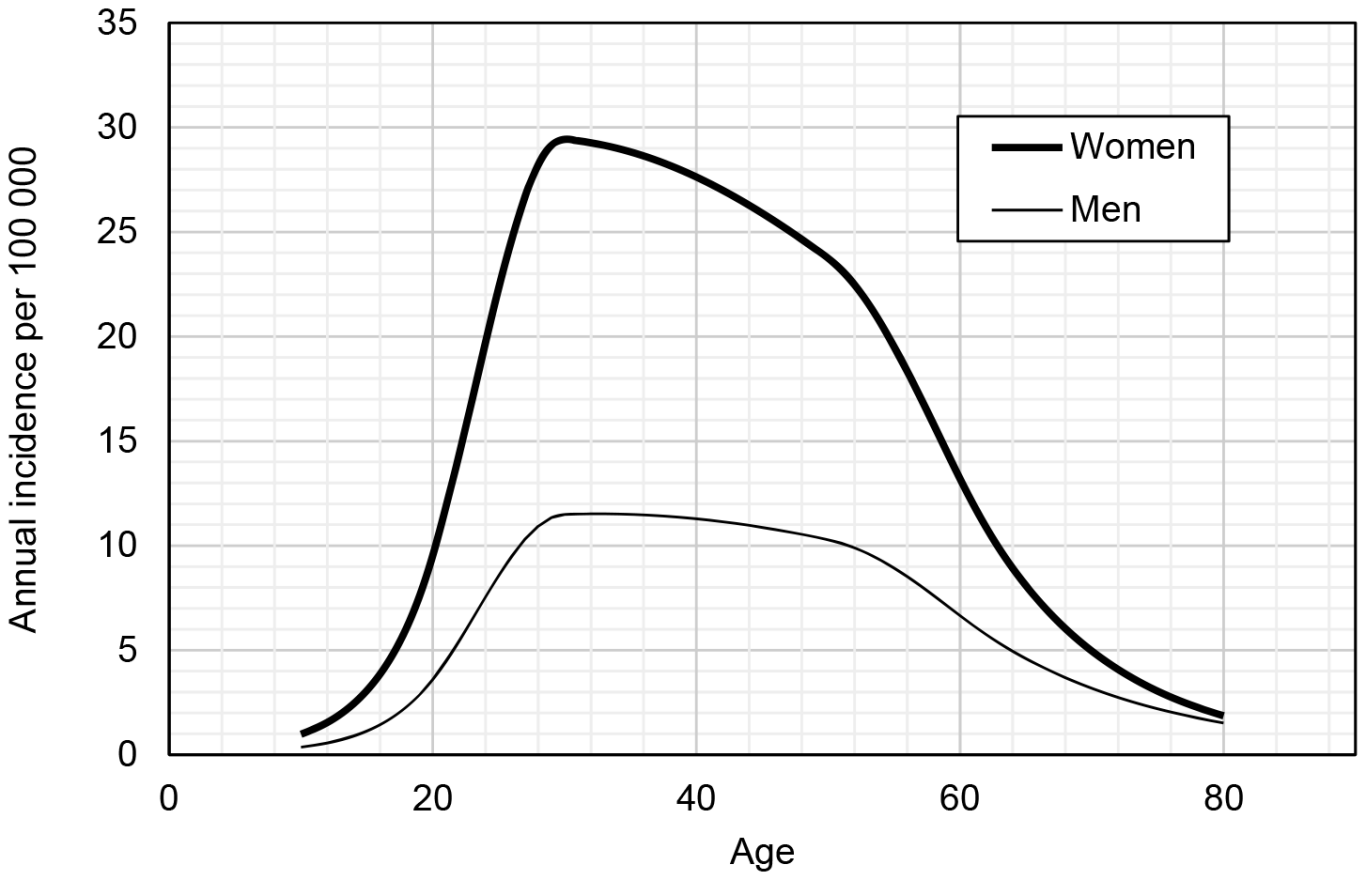
Hazard functions (incidence curves) of MS diagnosis estimated with spline functions.

**Figure 2:**
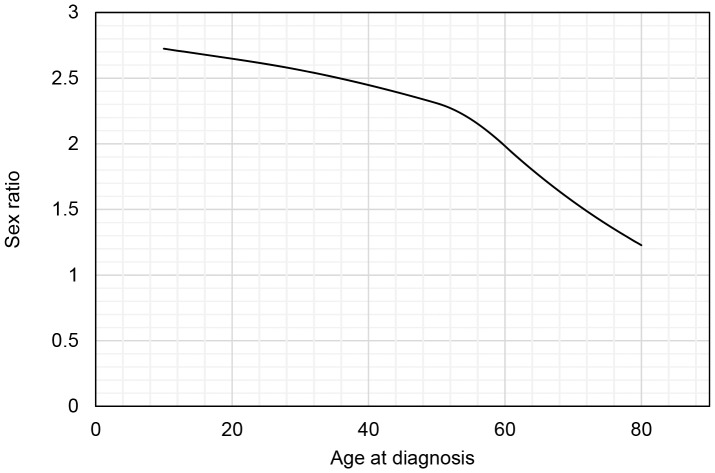
Female to male ratio at MS diagnosis. The hazard function is concave which implies that the mean of the ratio is less than the ratio at the mean age at diagnosis.

From 2001 to 2008 the age- and gender-specific hazard functions for the risk or incidence of a diagnosis of MS in Sweden was calculated (Figure 1). The annual MS incidence for females peeked at the age of 30 years and for males at 33 years with an annual incidence of 29.4 and 11.5 per 100,000 respectively. The female to male ratio decreased by age and was 2.65 at the age 20 years, 2.56 at 30 years and 2.45 at 40 years (Figure 2).

## Discussion

The overall nationwide age- and gender-specific incidence of MS in Sweden from 2001 to 2008 was 10.2 (6.2 for men and 14.0 for women) per 100,000 person-years and the overall female to male sex ratio was 2.26. Although, the incidence was considerably higher than previous regional Swedish estimates; 4.3 in Gothenburg city (1984–1988) [11], 5.2 in Västerbotten county (1988–1997) [10], and 6.4 in Värmland county (1991–2000) [9], we did not observe any change of MS incidence or sex-ratio during the incidence period.

Several studies [1]–[5] and systematic reviews [6], [7] report increased incidence of MS, primarily in women. However, besides the true change of MS frequency, higher MS prevalence in current compared with previous investigations may be explained by several other methodological factors. Although MS incidence is not affected by the survival time and better reflect MS risk, the number of cases and the observational time are often limited, making incidence estimates statistically uncertain. We had the privilege of using the Swedish nationwide mandatory and complete NPR registers with dated information about every clinical visit and discharge diagnoses as well as yearly information about place of residence for each patient, and annual gender-specific population figures in one-year age groups. Further advantages were the long observation period of 8 years, and the combination of data from two essentially independent sources, the SMSreg and the NPR. The mutual independence between the affiliation with the NPR and the SMSreg was a basic prerequisite of our method to calculate the MS incidence.

The low number of MS patients who were registered in the SMSreg only (n = 77, 0,3%) supports a high completeness of registered incident MS cases between 2001 and 2008 in the NPR. The registration rate was probably higher than that reported in the Danish national patient registry Landspatientregistret (LPR) [20] which share several features with the NPR. Although registration in both registries are mandatory, the LPR is hospital based, while the NPR includes all in- and outpatient care in Sweden. This difference might explain a completeness of only 92,8% in the LPR, compared with the Danish MS registry [21].

The MS diagnoses had not been validated in the NPR. However, there were conditions during the incidence period that favour diagnostic conformity. First; a neurologist almost always determines the diagnosis of MS in Sweden; second, the introduction of the McDonald criteria in 2001 [17] coincided with the start of our survey and the departments of neurology are expected to use the current diagnostic criteria; third, the availability of MRI for the diagnostic work-up is well distributed in Sweden. The validity of the MS diagnoses was 96,3% [21] in the Danish LPR. Since similar conditions prevail for registration of MS in the NPR, it is reasonable to assume that the validity of MS diagnosis is high also in the NPR. Contrary to the NPR, the SMSreg also included patients registered with Possible MS. Where the transition from Possible MS to MS in the SMSreg might have been delayed [17]–[19]; the diagnosis of MS in the NPR was registered after the diagnosis was confirmed. The proportion of Possible MS patients who were included in the present incidence estimation without a confirmed diagnosis in the NPR was small (less than 1%) and could therefore be ignored.

The NPR does not record whether a diagnosis is old or newly established. In order to estimate the MS incidence, we had to solve the problem of the probable date of MS diagnosis, as we did not know whether the first date of registration in the NPR was the date of MS diagnosis. We used the subset A, consisting of 5,241 patients for whom we had a date of diagnosis within the incidence period in the NPR, and a date of diagnosis in the SMSreg. Individually calculated probabilities of MS diagnosis from this subset of patients were based on age at the earliest date of diagnosis and time elapsed since 2001, and applied on other subsets of patients. The higher the age and the shorter the time elapsed since 2001, the lower the probability was that there was no earlier date of diagnosis before the incidence period. Thus, all individuals who might have been diagnosed with MS during the incidence period were included thanks to the use of probabilities in spite of incomplete information about earlier clinical visits. By using the logistic regression model, we avoided simplified rules and false assumptions, stating that MS diagnosis could not be before a certain time interval preceding the earliest date of an in- or outpatient care visit.

In other large-scale incidence surveys consisting of populations larger than one million persons, a number of challenging methodological problems had to be met. A high degree of ascertainment of MS patients may be achieved by repeat studies [12], [13], identifying patients from high coverage hospital registers [12], using health database and referral patterns [13] or by a national health insurance database [15]. In the UK, Alonso et al. [14] used the General Practice Research Database (GPRD) as a source for the patient population as well as the population at risk. The observed number of MS patients with a medical record in the GPRD was extended by the expected number, derived from the proportion of patients without a record. The incidence of MS was then generalized to the entire population of the UK [14]. In France, Fromont et al. [16] used the main French health insurance system, which covered 87% of the population of France, to investigate the nationwide incidence of MS. The number of new MS patients was approximated from the number of notifications for MS to this insurance system. The estimated MS incidence was generalized to the entire French population. Because the insurance system was the only source, Fromont et al. also accounted for an underestimation.

The overall MS incidence estimates reported in these large-scale studies were lower than the incidence in Sweden [12]–[16]. Lacking age-specific numbers of patients and population at risk, however, make accurate comparisons difficult. This also concerns the previous regional Swedish MS incidence estimates [9]–[11]. Our nationwide estimate showed 1.6–2.4 times higher MS incidence than previous regional surveys. However, they had not the possibility to use the Inpatient NPR or the SMSreg, and were performed before the start of the Outpatient NPR in 2001, making a more complete patient ascertainment difficult. Nevertheless, because of the magnitude of the increase in MS incidence, we cannot rule out that a real increase has occurred in Sweden.

The female/male sex ratio is considered a more robust epidemiological variable compared to prevalence and incidence, and less influenced by confounding factors, in particular if used by grouping patients by the year-of-birth approach [5], [22]. Several recent studies show an increase over time [5], [23]–[26] in particular confined to relapsing-remitting course [26]–[28] and influenced by latitudinal gradient [26], while others show stable sex ratios [29], [30]. An increase of sex ratio has also been reported in Scandinavia [27], [31]. Although, the sex ratio varied from 1.99 to 2.70 in Sweden 2001–2008 with an average of 2.26, there was no increase over time. In previous Swedish regional surveys of MS incidence the average female/male sex ratio was 1.45 (1950–1964) and 1.57 (1974–1988) in Gothenburg [11], 1.92 (1988–1997) in Västerbotten [10], and 2.50 (1991–1995) and 2.26 (1996–2000) in Värmland [9]. However, this temporal increase in MS female/male sex ratio was not confirmed in a nationwide investigation using the Swedish MS Register [32]. The average sex ratio was 2.62 estimated by year of birth and 2.57 by year of MS onset between 1931 and 1985. Thus, our estimate does not support a temporal increase of female/male sex ratio.

In conclusion, we present a new method for investigating MS incidence that could be applied on other populations. In supplement S2 we present how our tool could be applied for examining excess MS risk in different study materials: a cluster of MS, comparison of MS risk in different populations, and predicting MS risk in a population where the MS incidence is known. The nationwide Swedish MS incidence is one of the highest so far reported. Our data show considerably higher MS incidence than previously reported but do not support an increase of female/male sex ratio that have been claimed over recent years.

## Supporting Information

Supplement S1 **Logistic regression analysis and calculating the expected number of MS diagnosis.**
(DOCX)

Supplement S2 **The BASIC program is provided for calculating hazard functions.** Examples of how the method is applied for examining excess MS risk in different study materials: a cluster of MS, comparison of MS risk in different populations, and predicting MS risk in a population where the MS incidence is known.
(DOCX)
