# Supplementary material for: High Nationwide Incidence of Multiple Sclerosis in Sweden
Source: PLoS One. 2014 Sep 29;9(9):e108599. doi: 10.1371/journal.pone.0108599 (PMC4180935; doi:10.1371/journal.pone.0108599)
Supplement: Supplement S1 — Logistic regression analysis and calculating the expected number of MS diagnosis. (DOCX) [file pone.0108599.s001.docx]

***Supplement S1***

**Logistic regression analysis**

The unit for Age and Time since 1 January 2001was year (expressed with decimals). If a patient was included in subset C or H (Table 1), the age of the patient at the earliest date of diagnosis in the NPR and the time since 1 January 2001 to the earliest date of diagnosis in the NPR was calculated. Data were entered into the logistic model. Q(1), ... , Q(7) were set as the variable values (Q(0)=1 ). We calculated the Probability = β_0_ ⋅ Q(0)+ β_1_⋅ Q(1) + … + β_7_⋅ Q(7) for each individual. The Probability of the earliest date of diagnosis being on 1 January 2001 or later was 1/(1 + exp(-S)). Sex had no significant impact on the probability.

| Logistic regression coefficients | | | |
| --- | --- | --- | --- |
| **Variables** | **β** | **SE** | **p Value** |
| Constant | 2.59509 | 0.58962 | 0.0000 |
| Min(Age, 30) | -0.11975 | 0.02111 | 0.0000 |
| Max(Min(age-30, 50-30), 0) | -0.05278 | 0.00646 | 0.0000 |
| Max(Age-50, 0) | -0.07154 | 0.01007 | 0.0000 |
| Min(Time since 1 January 2001, 2) | 1.28386 | 0.07154 | 0.0000 |
| Max(Min(Time-2, 4-2), 0) | 0.63326 | 0.08680 | 0.0000 |
| Max(Min(Time-4, 6-4), 0) | 0.19068 | 0.11999 | 0.1121 |
| Max(Time-6, 0) | 0.07519 | 0.18887 | 0.6906 |

**Calculating the expected number of MS diagnosis**

The Poisson regression yielded the Beta coefficients. By use of the coefficients the expected number of MS diagnosis can be calculated. If the follow-up period of an individual starts at the age a and ends at b, then the contribution to the expected number equals the area under the incidence curve from a to b for the individual, i.e. the integral from a to b. By adding the contribution of all individuals in the group we get the expected number.

| Beta coefficients of the hazard functions for MS incidence | | | | | |
| --- | --- | --- | --- | --- | --- |
|  | **Variable** | **β** | **SE** | **HR (95%CI)** | **p Value** |
| **Men** | Constant | -14.8286 | 0.3734 |  |  |
|  | Q(2) | 0.2299 | 0.0166 | 1.26 (1.22-1.30) | 0.0000 |
|  | Q(3) | -0.0114 | 0.0011 | 0.99 (0.99-0.99) | 0.0000 |
|  | Q(4) | -0.0004 | 0.0004 | 1.00 (1.00-1.00) | 0.2929 |
|  | Q(5) | -0.0030 | 0.0006 | 1.00 (1.00-1.00) | 0.0000 |
|  |  |  |  |  |  |
| **Women** | Constant | -13.7981 | 0.2344 |  |  |
|  | Q(2) | 0.2271 | 0.0105 | 1.25 (1.23-1.28) | 0.0000 |
|  | Q(3) | -0.0114 | 0.0007 | 0.99 (0.99-0.99) | 0.0000 |
|  | Q(4) | -0.0004 | 0.0002 | 1.00 (1.00-1.00) | 0.0607 |
|  | Q(5) | -0.0039 | 0.0004 | 1.00 (1.00-1.00) | 0.0000 |

The definitions of the functions Q(i), i=1 to 5, are given below.

The spline functions may have knots at the ages 20,…, 60 years.

G(1)=20:G(2)=30:G(3)=50:G(4)=60

We put

Q(1)=1

and define some functions of age

Z(1)=Min(age, G(1))

Z(2)=Max(Min(age-G(1), G(2)-G(1), 0)

Z(3)=Max(Min(age-G(2), G(3)-G(2), 0)

Z(4)=Max(Min(age-G(3), G(4)-G(3), 0)

Z(5)=Max(age-G(4), 0)

Then the definitions of Q(i) are given by

Q(2)=Z(1)+Z(2)+Z(3)+Z(4)+Z(5)

Q(3)=Z(2)^2^+2⋅(G(2)-G(1))⋅(Z(3)+Z(4)+Z(5))

Q(4)= Z(3)^2^+2⋅(G(3)-G(2))⋅(Z(4)+Z(5))

Q(5)= Z(4)^2^+2⋅(G(4)-G(3))⋅Z(5)

The hazard function is exp(Σ βi Q(i)). More details are given for the calculation in the supplement 2.
