# Supplementary material for: High Nationwide Incidence of Multiple Sclerosis in Sweden
Source: PLoS One. 2014 Sep 29;9(9):e108599. doi: 10.1371/journal.pone.0108599 (PMC4180935; doi:10.1371/journal.pone.0108599)
Supplement: Supplement S2 — The BASIC program is provided for calculating hazard functions. Examples of how the method is applied for examining excess MS risk in different study materials: a cluster of MS, comparison of MS risk in different populations, and predicting MS risk in a population where the MS incidence is known. (DOCX) [file pone.0108599.s002.docx]

***Supplement S2***

The following BASIC program calculates the area under the hazard function for a woman from the ages of 10 years to 45 years. For other age intervals and sex (man=0, woman=1), obvious changes have to be done at line 90.

In general it is easy for a programmer to translate the program to another language.

10 DEF FNMIN(A,B)=(A+B-ABS(A-B))/2

20 DEF FNMAX(A,B)=(A+B+ABS(A-B))/2

30 DIM T(1,5)

40 G(1)=20:G(2)=30:G(3)=50:G(4)=60

50 FOR SEX=0 TO 1:FOR J=1 TO 5:READ T(SEX,J):NEXT J: NEXT SEX

60 DATA -14.8286, 0.2299,-0.0114,-0.000378,-0.0030

70 DATA -13.7981, 0.2271,-0.0114,-0.000443,-0.0039

80 Q(1)=1

90 SEX=1:A=10:B=45:REM A woman is followed from 10 to 45 years of age

100 FOR AGE=A TO B STEP .1

130 Z(1)=FNMIN(AGE, G(1))

140 Z(2)=FNMAX(FNMIN(AGE-G(1), G(2)-G(1)), 0)

150 Z(3)=FNMAX(FNMIN(AGE-G(2), G(3)-G(2)), 0)

160 Z(4)=FNMAX(FNMIN(AGE-G(3), G(4)-G(3)), 0)

170 Z(5)=FNMAX(AGE-G(4), 0)

180 Q(2)=Z(1)+Z(2)+Z(3)+Z(4)+Z(5)

190 Q(3)=Z(2)^2+2*(G(2)-G(1))*(Z(3)+Z(4)+Z(5))

200 Q(4)= Z(3)^2+2*(G(3)-G(2))*(Z(4)+Z(5))

210 Q(5)= Z(4)^2+2*(G(4)-G(3))*(Z(5))

230 L=0

240 FOR J=1 TO 5

250 L=L+Q(J)*T(SEX,J)

260 NEXT J

270 SUM=SUM+EXP(L)*.1

280 NEXT AGE

290 PRINT SUM:REM SUM is the area under the curve

In order to simplify the description of how the program can be modified to other applications, we have made some jumps in the enumerations of the lines.

**Application of the method**

***A cluster.***

Haahr *et al*[^10^](#_ENREF_10) described a cluster of 8 MS patients who for 7 years had attended the same school in Denmark. By use of the hazard functions we could calculate the expected number of patients with a diagnosis of MS if the risk coincided with that of Sweden.

The contribution to the risk starts at 10 years. The expected numbers SUM(0) and SUM(1) of men (=0) and women (=1), respectively, with a MS diagnosis can be calculated by the modified version of the program.

Line 90 in the program should be exchanged for the following lines

90 FOR R=1 TO 13

100 READ N

110 DATA 13,10,9,12,12,13,13,12,9,10,12,10,8

120 FOR AGE=10 TO 52-R

The lines at the end (after line 210) should be exchanged for

220 FOR SEX=0 TO 1

230 L=0

240 FOR J=1 TO 5

250 L=L+Q(J)*T(SEX,J)

260 NEXT J

270 SUM(SEX)=SUM(SEX)+EXP(L)*N/2:REM With the probability 1/2 we assume that the pupil is a girl

280 NEXT SEX

290 NEXT AGE

300 NEXT R

310 PRINT SUM(0),SUM(1)

The hazard ratio of 11.54 was calculated as the quotient 8/0.6929. If Z has a Poisson distribution with the mean 0.6929, then the probability that Z is larger than or equal to 8 equals 6⋅10^−7^ and 1/(6⋅10^−7^ ) = 1.7⋅10^6^ . The 95% confidence interval (CI) of the hazard ratio was calculated exactly by use of Poisson distribution. Thus 1 school of 1.7 million schools of the same size (145 pupils) was expected to have 8 or more cases.

*Reference.* Haahr, S, Munch, M, Christensen, T*,* et al*.* Cluster of multiple sclerosis patients from Danish community. *Lancet*. 1997;349(9056):923.

***Comparison of MS risk in a certain population with that in Swede****n.*

*Eliasdottir et al.* reported an overall age- and gender-specific incidence of diagnosis of MS in Iceland during the period from 2002 to 2007 at 7.6 per 100,000 (3.8 for men, 11.5 for women) and, when adjusted to the 2000 US white population, as 8.2 per 100,000. Virtually complete case ascertainment was achieved in the approximately 300,000 Icelandic population. The number of new MS patients during the incidence period (1,781,102 person-years) was 136 (34 men and 102 women). The age- and gender-specific numbers of both patients and person-years for observation were tabulated in 5-year age groups. The MS incidence was also shown as curves by age in 5-year age groups for each gender. We compared the MS incidence reported from Iceland with that of Sweden.

For the calculation of the expected numbers, the following modifications were performed. Line 90 was exchanged for

90 OPEN "I",1,"ISLAND.TXT"

100 FOR R=1 TO 14

110 INPUT#1,A$,AGE,N(0),N(1):IF R=1 THEN N(0)=N(0)/3:N(1)=N(1)/3

The file, ISLAND.TXT gives the population numbers for men and women and looked like the following:

0–14,12,200890,193072

15–19,17, 66566,63439

20–24,22, 67004,64775

25–29,27, 66529,64022

30–34,32, 65515,61920

35–39,37, 64595,62460

40–44,42, 66804,64998

45–49,47, 64722,61121

50–54,52, 57151,53977

55–59,57, 47928,45386

60–64,62, 35268,35270

65–69,67, 27410,28777

70–74,72, 25269,28122

75–79,77, 20116,24273

At the end of the program (after line 210), we exchanged the lines for:

220 FOR SEX=0 TO 1

230 L=0

240 FOR J=1 TO 5

250 L=L+Q(J)*T(SEX,J)

260 NEXT J

270 SUM(SEX)=SUM(SEX)+EXP(L)*N(SEX)

280 NEXT SEX

290 NEXT R

300 PRINT SUM(0),SUM(1)

The age- and gender-specific incidence of MS diagnosis in Iceland during the period from 2002 to 2007 was significantly lower than that in Sweden from 2001 to 2008. For men, the expected number if the risk coincided with that in Sweden was 55.07 and the observed number was 34 (p=0.0031). The hazard ratio was 0.62, 95% CI 0.43–0.86. For women the expected number if the risk coincided with that in Sweden was 128.62 and the observed number was 102 (p=0.0088). The hazard ratio was 0.79, 95% CI 0.65–0.96.

*Reference.* Eliasdottir, OJ, Olafsson, E, Kjartansson, O. Incidence of multiple sclerosis in Iceland, 2002–2007: a population-based study. *Mult Scler*. 2011;17(8):909–13.

***Prediction of MS risk in a population where the MS incidence is known.***

As a result of the present study (Ahlgren et al.), the age- and gender-specific incidence of diagnosis of MS in Gothenburg, Sweden during 2001 to 2008 was known.

We calculated the number of new patients diagnosed with MS in the year 2011 in Gothenburg.

Line 90 in the program should be exchanged for the following lines

90 OPEN "I",1,"GBG.CSV"

100 LINE INPUT#1,F$

92 DIM N(1,100)

110 FOR AGE=0 TO 100:INPUT#1,A$,B$,C$,N(0,AGE),N(1,AGE):NEXT AGE

112 CLOSE 1

120 FOR AGE=10 TO 80

For the calculation, we needed the population figures for men and women.

The beginning of the file GBG.CSV gives the population numbers, and looks like the following:

Age,Year,Region,men,women

0 year,2011,Göteborg,3731,3553

1 year,2011,Göteborg,3683,3598

2 year,2011,Göteborg,3390,3349

3 year,2011,Göteborg,3247,3027

4 year,2011,Göteborg,2999,2910

5 year,2011,Göteborg,2947,2843

6 year,2011,Göteborg,2703,2625

7 year,2011,Göteborg,2707,2473

8 year,2011,Göteborg,2587,2518

9 year,2011,Göteborg,2367,2425

10 year,2011,Göteborg,2432,2290

11 year,2011,Göteborg,2396,2211

12 year,2011,Göteborg,2328,2169

13 year,2011,Göteborg,2463,2289

The lines at the end (after line 210) should be exchanged for:

220 FOR SEX=0 TO 1

230 L=0

240 FOR J=1 TO 5

250 L=L+Q(J)*T(SEX,J)

260 NEXT J

270 SUM(SEX)=SUM(SEX)+EXP(L)*N(SEX,AGE)

280 NEXT SEX

290 NEXT AGE

300 PRINT SUM(0),SUM(1)

The expected number of patients diagnosed with MS in Gothenburg in 2011 would be 57.5 (17.2 men and 40.3 women) with a 95% tolerance interval of 42 to 74. If the number of observed patients diagnosed with MS in Gothenburg in 2011 is on the borders of or outside this interval, it would indicate that the MS risk significantly differs from that in Sweden overall from 2001 to 2008.

With similarly high precision, a possible excess or low MS risk in, for example, women before, during, and after pregnancy or in patients with another autoimmune disease (such as Type 1 Diabetes or Inflammatory Bowel Disease) can be estimated.
